# Central lymph node ratio predicting lateral metastasis in pediatric thyroid cancer: a retrospective study

**DOI:** 10.3389/fendo.2026.1787834

**Published:** 2026-04-27

**Authors:** Xiaoming Wang, Xinyu Zhao, Yuhang Deng, Jiaojiao Zhao, Tianxiang Cui, Qianhou Huang, Guoyang Wu, Xiubo Lu

**Affiliations:** 1 Department of Thyroid Surgery, The First Affiliated Hospital of Zhengzhou University, Zhengzhou, Henan, China; 2 Xiamen Medical College Affiliated Haicang Hospital, Xiamen, Fujian, China

**Keywords:** central lymph node ratio, differentiated thyroid carcinoma, lateral lymph node metastasis, pediatric, predictive factors, predictive model

## Abstract

**Purpose:**

To explore whether central lymph node ratio (LNR) can be used to predict the lateral lymph node metastasis (LLNM) in pediatric patients with differentiated thyroid carcinoma (DTC) and construct a predictive model.

**Methods:**

We reviewed the clinicopathological data of patients with DTC (aged ≤14 years) who had thyroid lobectomy (unilateral or bilateral)and therapeutic central neck dissection (CND) with concurrent or staged lateral neck dissection (LND) from 2015 to 2025. Patients were grouped by LLNM status. Receiver Operating Characteristic Curve (ROC) analysis identified the optimal LNR cutoff.

**Results:**

In univariate analysis, central LNR threshold of 0.4 predicts LLNM with a sensitivity of 90.0%, specificity of 76.0%, and OR of 11.374.Multivariate analysis identified central LNR (OR = 3.741), bilaterality (OR=3.850), and ≥4 metastatic central nodes (OR = 4.732) as independent predictors.

**Conclusion:**

Central LNR, bilaterality, and ≥4 metastatic central lymph nodes were independent predictors. An LNR ≥0.4 serves as a key indicator for identifying high-risk pediatric DTC patients.

## 1 Introduction

Thyroid cancer is a rare form of malignant tumor in children and adolescents, with differentiated thyroid carcinoma (DTC) being the most widespread type. The annual incidence of pediatric DTC ranges from 0.5 to 10 cases per 100,000 individuals (1). Compared to adult thyroid cancer, pediatric and adolescent thyroid cancer (hereafter referred to as “pediatric thyroid cancer”) exhibits distinct biological behaviors including typically, larger primary tumors, higher rates of lymph node metastasis, and more advanced TNM stages at diagnosis (2). However, long-term survival rates are relatively excellent. This characteristic of “high metastasis, high recurrence, low mortality” makes the treatment strategy for pediatric thyroid cancer, particularly the determination of the extent of surgery, a focus of clinical controversy. Presently, surgery remains the primary treatment modality. For clinically confirmed (cN1) lateral neck lymph node metastasis, therapeutic lateral neck lymph node dissection (LND) should be performed (3–5). However, significant disagreement remains regarding whether prophylactic lateral neck dissection (PLND) should be carried out on clinically and radiologically negative (cN0) lateral necks (6–8). Overly extensive dissection is associated with an enhanced risk of surgical complications, including spinal accessory nerve injury, cervical plexus nerve injury, chyle leak, and hypoparathyroidism (9–11), severely impacting the child’s quality of life. Conversely, failing to address lateral compartment metastasis in at-risk patients may lead to residual disease, postoperative recurrence, and the need for reoperation, increasing treatment difficulty and physical and mental burden, potentially affecting the child’s growth and development.

Therefore, identifying an accurate predictor of lateral lymph node metastasis is crucial for developing individualized and optimized surgical plans. According to previous studies, current predictors for lateral lymph node metastasis (LLNM) include tumor size, multifocality, TSH ≥2.910, tumor located in the upper pole, extracapsular invasion, number of central lymph node metastasis(CLNM), and positive CLNM (12–14). Among these, a central LNR exceeding 77.78% was identified as a significant predictor of recurrence in pediatric differentiated thyroid carcinoma (DTC) patients under 19 years old (15). However, research specifically targeting the predictive value of central lymph node ratio (CLNR) for lateral compartment LNM in the distinct pediatric population, particularly young children, is insufficient and lacks specificity. Based on this background, this study aims, through a retrospective analysis, to systematically investigate for the first time the clinical value of CLNR in predicting lateral LNM in pediatric DTC patients. It further evaluates the validity of predictive models to provide novel, more reliable quantitative tools for making clinical choices.

## 2 Population and methods

### 2.1 Population involved in the research

The design of this study was retrospective and cohort based. Clinical data were consecutively collected from all pediatric DTC patients (age ≤14 years) who underwent surgical treatment in the Department of Thyroid Surgery at our hospital between January 2015 and January 2025.

Inclusion criteria: 1. Pathologically confirmed DTC postoperatively; 2. Age ≤14 years; 3. Initial surgery performed at our hospital; 4. Surgical procedure involving thyroid lobectomy (unilateral or bilateral) as well as therapeutic central neck dissection (CND); 5. Underwent concurrent or staged therapeutic lateral neck dissection (levels II-V); 6. Complete clinicopathological data available.

Exclusion criteria: (1) Any previous history of surgery on the thyroid; (2) Concurrent other head and neck malignancies or history of head and neck radiotherapy; (3) Pathological diagnosis of non-differentiated thyroid carcinoma; (4) Incomplete clinical data.

Finally, 148 patients were included in this study.

### 2.2 Surgical methods and data collection

The central lymph node dissection for all surgeries included the pre-laryngeal, pre-tracheal, and bilateral paratracheal lymph nodes (level VI). Lateral neck dissection followed the principles of systematic functional neck dissection, covering levels IIa, IIb, III, IV, and V (16, 17). For patients undergoing unilateral thyroid lobectomy, only the ipsilateral paratracheal lymph nodes (Region VI) were routinely dissected, with no involvement of the contralateral side. For patients undergoing bilateral thyroid lobectomy, bilateral paratracheal lymph nodes (Region VI) were routinely dissected. Among the patients included in this study, there were no cases of isolated dissection of the contralateral paratracheal lymph nodes (Region VI).

Clearing of the seventh segment (superior mediastinum) was not a routine procedure; it is performed only in patients with widespread metastasis to central lymph nodes, such as when preoperative imaging indicates involvement. None of the patients who underwent CND in this study met the criteria for Level VII dissection.

Routine lateral neck dissection covered levels IIa, III, and IV. Level IIb was examined only intraoperatively if suspicious. Level VA and IIb were not routinely dissected without preoperative imaging evidence.

Collected clinicopathological data included:

- Demographic data: Age, gender.
- Preoperative data: Family history, coexistence of Hashimoto’s thyroiditis, preoperative ultrasound grading, TSH, FT3, FT4, serum Tg.
- Postoperative pathological data: Surgical procedure, maximum tumor diameter, pathological type, TNM stage, multifocality, bilaterality, extrathyroidal extension (ETE), BRAF V600E mutation status, RAS family gene mutation status.
- Multifocal lesions were defined as the presence of at least two lesions in one lobe, or in both lobes.
- Extrathyroidal extension (ETE) was defined according to the American Joint Committee on Cancer (AJCC) 8th edition staging system as tumor extension into the perithyroidal soft tissues, including minimal (gross or microscopic) and extensive (invasion of surrounding structures) extension (18).
- Lymph node data: The total number of lymph nodes removed from the central region and the number of positive lymph nodes in this region. The total number of lymph nodes removed from the lateral neck region and the number of positive lymph nodes in this region.
- Calculated metric: CLNR= the number of metastatic central lymph nodes divided by the total number of lymph nodes removed from the central region.

### 2.3 Data statistical analysis

Data analysis was carried out using SPSS 26.0 software (29.0.2.0, IBM Corporation, Armonk, NY, USA) and R (version 4.4.1). Normality tests were first conducted on continuous variables. Variables following a normal distribution were reported as mean ± SD and compared using the independent samples t-test. Non-normally distributed variables were presented as median (interquartile range) [M (IQR)] and compared using the Mann-Whitney U test. Categorical variables were displayed as proportions (n (%)) and contrasted using either the chi-squared test or Fisher’s exact test (**Table 1**). The linearity of the relationships between continuous variables (number of positive central lymph nodes, CLNR, and tumor diameter) and the presence of LLNM were assessed using the Box-Tidwell test. The findings demonstrated a notable non-linear association between central LNR and LLNM (P = 0.024), while no significant non-linear effects were found for the number of positive central lymph nodes (CLNs) (P = 0.149) and tumor diameter (P = 0.793). However, given the relatively low P-value for the number of positive CLNs, ROC curve analyses were performed for both central LNR and the number of positive CLNs to evaluate their impact on LLNM. The optimal clinical cut-off values for predicting LLNM were determined based on the maximum Youden index. For central LNR, the optimal cut-off was 0.40, and patients were stratified into <0.4 and ≥0.4 groups. For the number of positive CLNs, the optimal cut-off was 3.5, adjusted to an integer of 4, and patients were stratified into <4 and ≥4 groups. (**Table 2**) The remaining continuous variable, tumor diameter, was included directly in the final multivariate model.

**Table 1:** Comparison of clinicopathological characteristics between pediatric DTC patients with and without lateral lymph node metastasis (LLNM).

Characteristic	Total (n=148)	LLNM+ (n=90)	LLNM- (n=58)	P-value
**Gender, n (%)**				0.449
Male	40 (27.0%)	22 (24.4%)	18 (31.0%)	
Female	108 (73.0%)	68 (75.6%)	40 (69.0%)	
**Age, median**	12 (10-13.75)	12(10-13.25)	13 (11-14)	0.338
**Histological subtype, n (%)**				0.151
PTC	141 (95.3%)	88 (97.8%)	53 (91.4%)	
FTC	5 (3.4%)	1 (1.1%)	4 (6.9%)	
MTC	2 (1.4%)	1 (1.1%)	1 (1.7%)	
**Hashimoto's Thyroiditis, n (%)**				1.000
Yes	31 (20.9%)	19 (21.1%)	12 (20.7%)	
No	117 (79.1%)	71 (78.9%)	46 (79.3%)	
**Tumor size (mm), median (IQR)**	18(8.00-27.75)	20(10.75-30.00)	10(7.00-20.00)	**0.020**
**TSH (μIU/mL), median (IQR)**	3.72(2.07-4.88)	4.88(2.50-5.46)	2.65(1.56-4.88)	**0.010**
**FT3 (pmol/L), median (IQR)**	6.10(5.51-6.31)	6.02(5.47-6.31)	6.25(5.60-6.31)	0.194
**FT4 (pmol/L), median (IQR)**	12.26(10.30-12.57)	11.73(10.20-12.27)	12.27(10.68-13.24)	0.330
**Serum Tg, median (IQR)**	206.25(25.30-206.25)	205.63(25.92-206.26	206.25(20.85-206.25)	0.555
**T stage, n (%)**				**<0.010**
T1a	53 (35.8%)	23 (25.6%)	30 (51.7%)	
T1b	39 (26.4%)	23 (25.6%)	16 (27.6%)	
T2	37 (25.0%)	30 (33.3%)	7 (12.1%)	
T3	13 (8.8%)	9 (10.0%)	4 (6.9%)	
T4	6 (4.1%)	5 (5.6%)	1 (1.7%)	
**N stage, n (%)**				**N/A**
N0	17 (11.5%)	0 (0.0%)	17 (29.3%)	
N1a	41 (27.7%)	0 (0.0%)	41 (70.7%)	
N1b	90 (60.8%)	90 (100.0%)	0 (0.0%)	
**M stage, n (%)**				0.707
M0	145 (98.0%)	87 (96.7%)	58 (100.0%)	
M1	3 (2.0%)	3 (3.3%)	0 (0.0%)	
**Multifocality, n (%)**				**0.041**
Yes	18 (12.2%)	15 (16.7%)	3 (5.2%)	
No	130 (87.8%)	75 (83.3%)	55 (94.8%)	
**Bilaterality, n (%)**				**<0.010**
Yes	51 (34.5%)	44 (48.9%)	7 (12.1%)	
No	97 (65.5%)	46 (51.1%)	51 (87.9%)	
**Extrathyroidal Extension, n (%)**				0.529
Yes	11 (7.4%)	8 (8.9%)	3 (5.2%)	
No	137 (92.6%)	82 (91.1%)	55 (94.8%)	
**No. of Positive CLNs, median (IQR)**	4 (1-9)	6 (3-11)	1 (0-3)	**<0.010**
**Central LNR, median (IQR)**	0.48 (0.08-0.84)	0.67 (0.41-0.90)	0.09 (0-0.39)	**<0.010**

Continuous variables are presented as median (IQR) and were compared using the Mann-Whitney U test; categorical variables are presented as n (%) and were compared using the chi-square or Fisher’s exact test. Bold values indicate statistical significance (P < 0.05).

**Table 2:** Central lymph node ratio (CLNR) and central lymph node metastasis (CLNM) count status stratified by LLNM status.

Characteristic	Total (n=148)	LLNM+ (n=90)	LLNM- (n=58)	P-value
**Central LNR Group, n (%)**				**<0.010**
LNR <0.4	64 (43.2%)	20 (22.2%)	44 (75.9%)	
LNR ≥0.4	84 (56.8%)	70 (77.8%)	14 (24.1%)	
**CLNM Count Status (Cutoff=4), n (%)**				**<0.010**
≥4	75 (50.7%)	65 (72.2%)	10 (17.2%)	
<4	73 (49.3%)	25 (27.8%)	48 (82.8%)	

Bold values indicate statistical significance (P < 0.05).

Variables with P < 0.10 in univariate analysis were entered into a multivariate logistic regression model using backward stepwise selection based on the likelihood ratio test, with an exclusion criterion of P > 0.10. The final model was determined by the likelihood ratio test. Predictive performance was evaluated using ROC curve analysis, and the area under the curve (AUC) was calculated for each predictor, especially for CLNR. A P-value of less than 0.05 was deemed statistically significant.

## 3 Results

**Figure 1:**
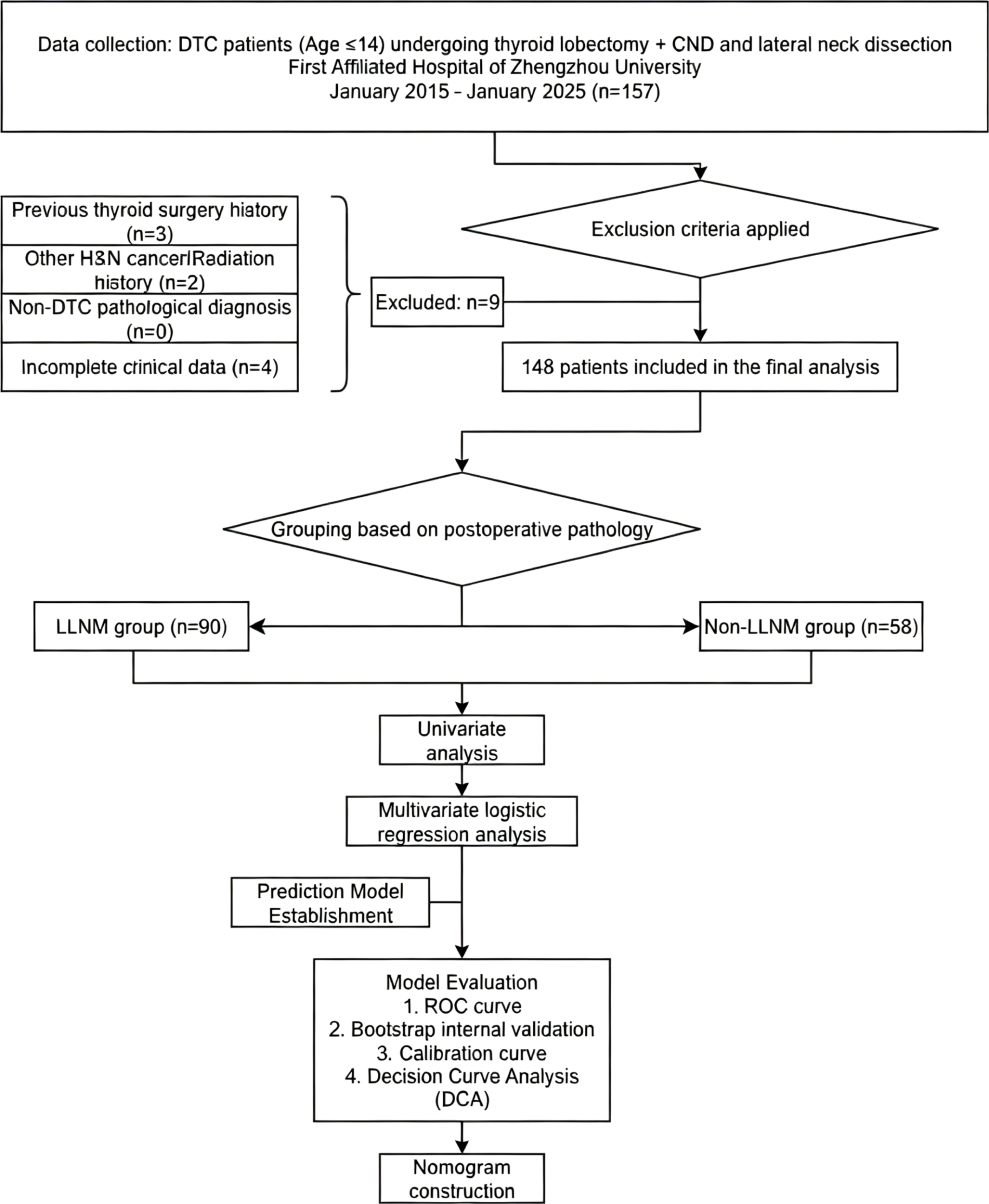
Flowchart of the patient selection process. The diagram outlines the inclusion and exclusion criteria, resulting in a final cohort of 148 pediatric DTC patients categorized by LLNM status.

Between January 2015 and January 2025, a cohort of 157 children underwent surgery for thyroid carcinoma. The study excluded nine patients who met the exclusion criteria. **Figure 1** presents a flowchart illustrating the process of selecting patients.

Totally,148 patients were recruited for the study, comprising 40 males and 108 females (male-to-female ratio 1:2.7). The median age is 12 years(IQR: 10-13.75). All patients underwent thyroid lobectomy (unilateral or bilateral) and lymph node dissection. The LLNM-positive group consisted of 90 patients (60.8%), and the LLNM-negative group consisted of 58 patients (39.2%). No significant differences in age or gender distribution were found between the two groups (P > 0.05). The baseline data for the patients are presented in **Table 3**.

**Table 3:** Baseline clinicopathological characteristics of the entire study cohort (N = 148).

Characteristic	Total (n=148)
Gender, n (%)	
Male	40 (27.0%)
Female	108 (73.0%)
**Age, median**	12 (10-13.75)
Histological subtype, n (%)	
PTC	141 (95.3%)
FTC	5 (3.4%)
MTC	2 (1.4%)
Hashimoto's Thyroiditis, n (%)	
Yes	31 (20.9%)
No	117 (79.1%)
**Tumor size (mm), median (IQR)**	18(8.00-27.75)
**TSH (μIU/mL), median (IQR)**	3.72(2.07-4.88)
**FT3 (pmol/L), median (IQR)**	6.10(5.51-6.31)
**FT4 (pmol/L), median (IQR)**	12.26(10.30-12.57)
**Serum Tg, median (IQR)**	206.25(25.30-206.25)
T stage, n (%)	
T1a	53 (35.8%)
T1b	39 (26.4%)
T2	37 (25.0%)
T3	13 (8.8%)
T4	6 (4.1%)
N stage, n (%)	
N0	17 (11.5%)
N1a	41 (27.7%)
N1b	90 (60.8%)
M stage, n (%)	
M0	145 (98.0%)
M1	3 (2.0%)
Multifocality, n (%)	
Yes	18 (12.2%)
No	130 (87.8%)
Bilaterality, n (%)	
Yes	51 (34.5%)
No	97 (65.5%)
Extrathyroidal Extension, n (%)	
Yes	11 (7.4%)
No	137 (92.6%)
**No. of Positive CLNs, median (IQR)**	4 (1-9)
**Central LNR, median (IQR)**	0.48 (0.08-0.84)
Central LNR Group, n (%)	
LNR <0.4	64 (43.2%)
LNR ≥0.4	84 (56.8%)
CLNM Count Status (Cutoff=4), n (%)	
≥4	75 (50.7%)
<4	73 (49.3%)

Continuous variables are shown as median (IQR) and categorical variables as n (%).

Bold values indicate statistical significance (P < 0.05).

Of the 148 patients, 95 (64.2%) underwent bilateral lobectomy, 53 (35.8%) unilateral lobectomy. Two patients (1.4%) later underwent a second-stage isolated lateral neck dissection. Among the 148 patients, 97 (65.5%) had concurrent lateral neck dissection at initial surgery; of these, 70 (72.2%) had CLNR ≥0.4 and 90 (92.8%) had LLNM. The two patients who had staged lateral neck dissection both had CLNR ≥0.4 and LLNM(**Table 4**).

**Table 4:** Surgical procedures and lateral neck dissection outcomes.

Variable	n (%)
Initial surgical procedure(n=148)	
Bilateral lobectomy	95 (64.2)
Unilateral lobectomy	53 (35.8)
Second-stage isolated lateral neck dissection	2 (1.4)
Lateral neck dissection among lobectomy patients(n = 148)	
Simultaneous lateral neck dissection	97 (65.5)
–With high-risk features (CLNR ≥ 0.4)*	70 (72.2)^#^
–Diagnosed with lateral neck lymph node metastasis	90 (92.8)^#^
Staged lateral neck dissection	2 (1.4)
–With high-risk features (CLNR ≥ 0.4)*	2 (100)^#^
–Diagnosed with lateral neck lymph node metastasis	2 (100)^#^

* High-risk features defined as CLNR ≥0.4.

# Percentages calculated within the respective lateral neck dissection subgroup.

### 3.1 High-risk factors for LLNM in children under 14 years of age with DTC

Univariate analysis showed that tumor size, TSH level, T stage, multifocality, bilaterality, number of positive central lymph nodes, and CLNR value were significantly different between the LLNM-positive and LLNM-negative groups (P < 0.05). No major differences were evident in terms of gender, age, histological subtype, coexistence of Hashimoto’s thyroiditis, FT3, FT4, serum Tg, distant metastasis (M stage), or ETE status between the two groups (**Table 1**).

### 3.2 Predictive model establishment and evaluation

Include the following statistically significant variables from the single-factor analysis in the multiple-factor analysis: tumor size, TSH, T stage, multifocality, bilateral involvement, CLN positive lymph node group(≥ 4 vs<4), and CLNR group(≥ 0.4 vs<0.4). Perform multivariate logistic regression analysis using backward stepwise selection (with an exclusion criterion of P > 0.10). After adjusting for confounding factors such as T stage and multifocality, LNR ≥0.4 and number of positive lymph nodes ≥4 remained independent risk factors for LLNM. The final model showed that CLNR group (OR = 3.741, 95% CI: 1.320-10.607, P = 0.013), CLN positive lymph node group (OR = 4.732, 95% CI: 1.640-13.649, P = 0.004), T stage (OR = 1.759, 95% CI: 1.176-2.633, P = 0.006), and bilaterality (OR =3.850, 95% CI: 1.339-11.073, P = 0.012) were found to be independent risk-predicting factors for LLNM. (**Table 5**).

**Table 5:** Multivariate logistic regression analysis of independent risk factors for lateral lymph node metastasis (LLNM).

Variables in the equation	B	S.E.	Wald	df	P-value	Odds Ratio (OR)	95% CI for OR
T stage	0.565	0.206	7.545	1	0.006^*^	1.759	1.176 – 2.633
Bilaterality (Yes)	1.348	0.539	6.256	1	0.012^*^	3.850	1.339 – 11.073
Central LNR Group (≥0.4)	1.319	0.532	6.160	1	0.013^*^	3.741	1.320 – 10.607
CLN Metastasis Count Group (≥4)	1.554	0.541	8.268	1	0.004^*^	4.732	1.640 – 13.649
Constant	-2.789	0.634	19.358	1	<0.001	0.062	

*P<0.05was considered statistically significant.

Variables entered in the initial model: tumor size, TSH, T stage, multifocality, bilaterality, CLNR group, and positive CLN count group. Final model was derived using backward stepwise selection (exclusion criterion>0.10). OR,odds ratio; CI, confidence interval. Bold values indicate statistical significance (P < 0.05).

**Figure 2:**
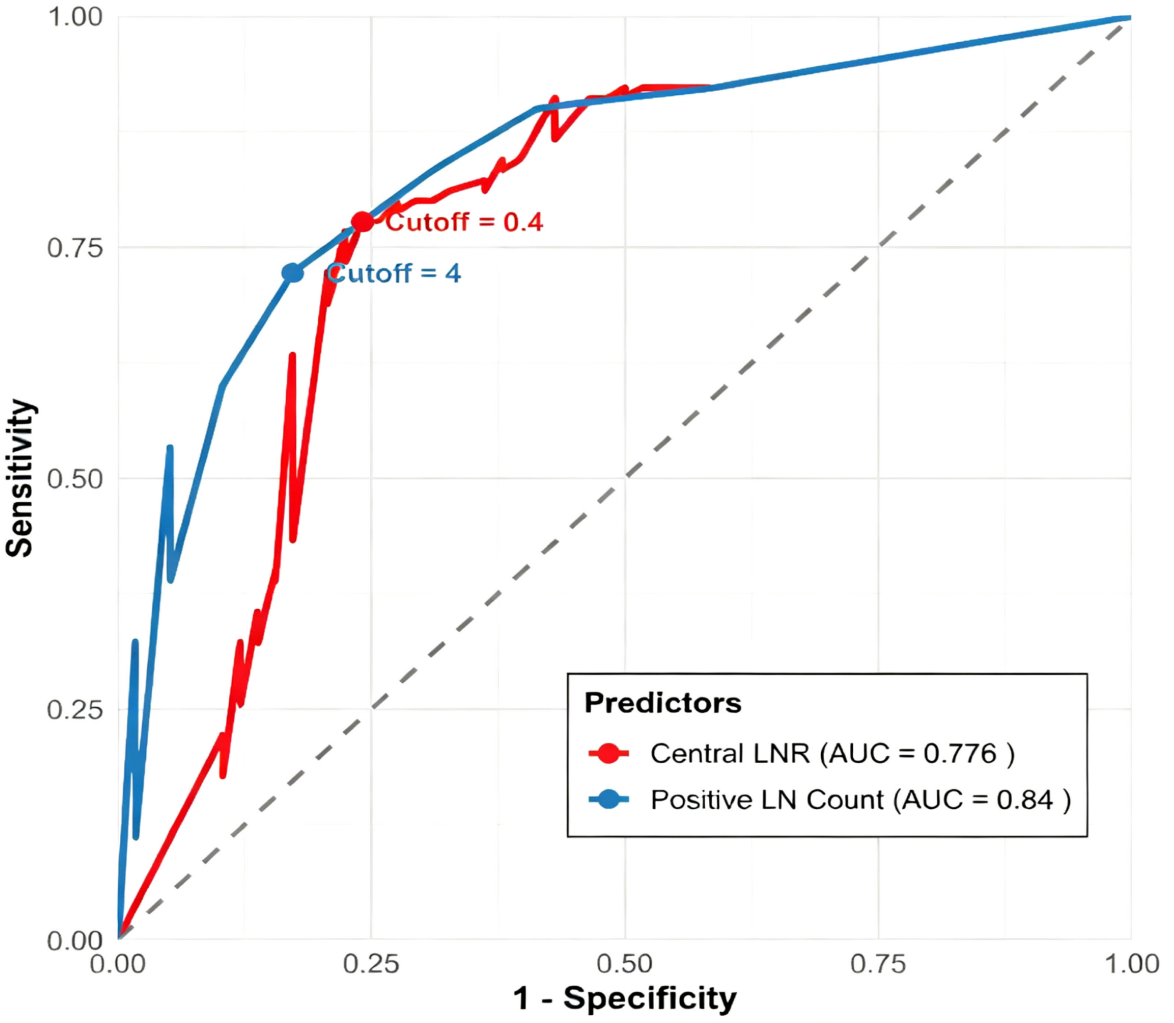
ROC curves for individual predictors. Receiver operating characteristic (ROC) curves of central lymph node ratio (CLNR, AUC = 0.776) and number of positive lymph nodes (AUC = 0.84), identifying optimal cutoffs of 0.4 and 4, respectively.

**Figure 3:**
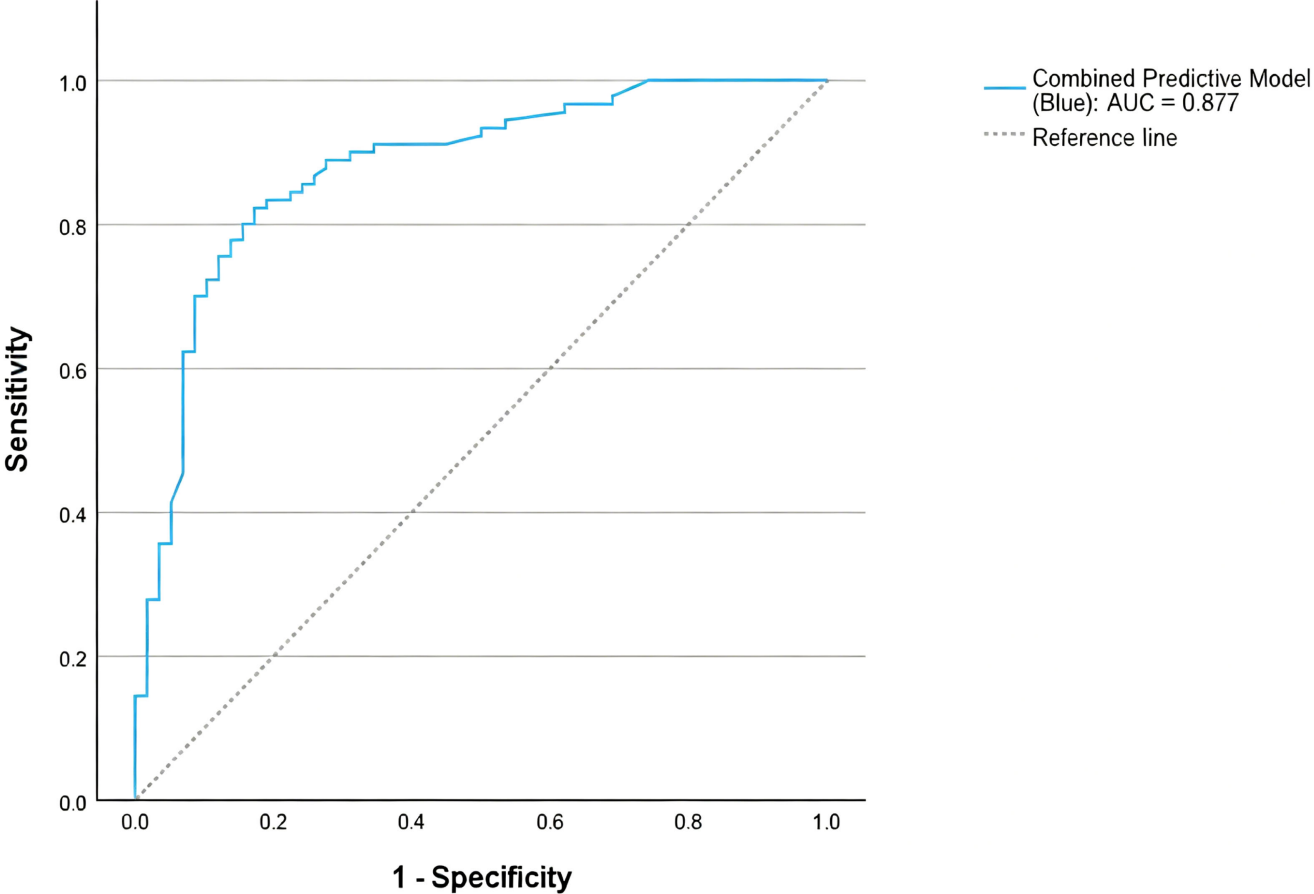
ROC curve of the combined predictive model. The integrated model, incorporating T stage, bilaterality, CLNR, and positive CLN count, achieved an AUC of 0.877.

The model’s predictive ability was indicated by an AUC of 0.877, significantly higher than any single indicator(**Figure 2**), demonstrating the model’s excellent discriminative ability for the presence or absence of lateral regional lymph node metastasis (**Figure 3**).

### 3.3 Bootstrap internal validation

**Figure 4:**
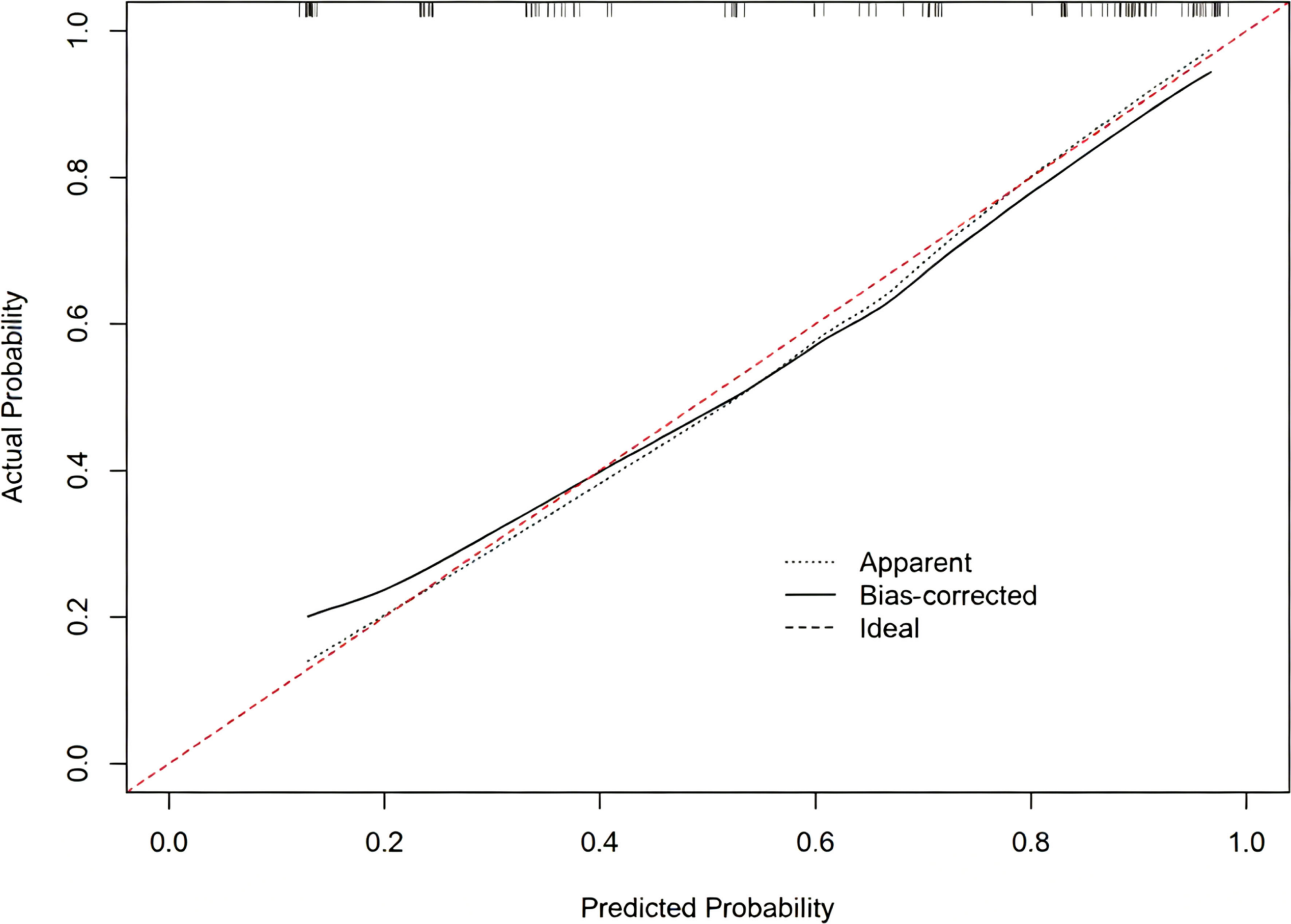
Calibration plot of the predictive model. The plot illustrates the agreement between predicted and observed probabilities of LLNM. The dotted line represents ideal performance.

**Figure 5:**
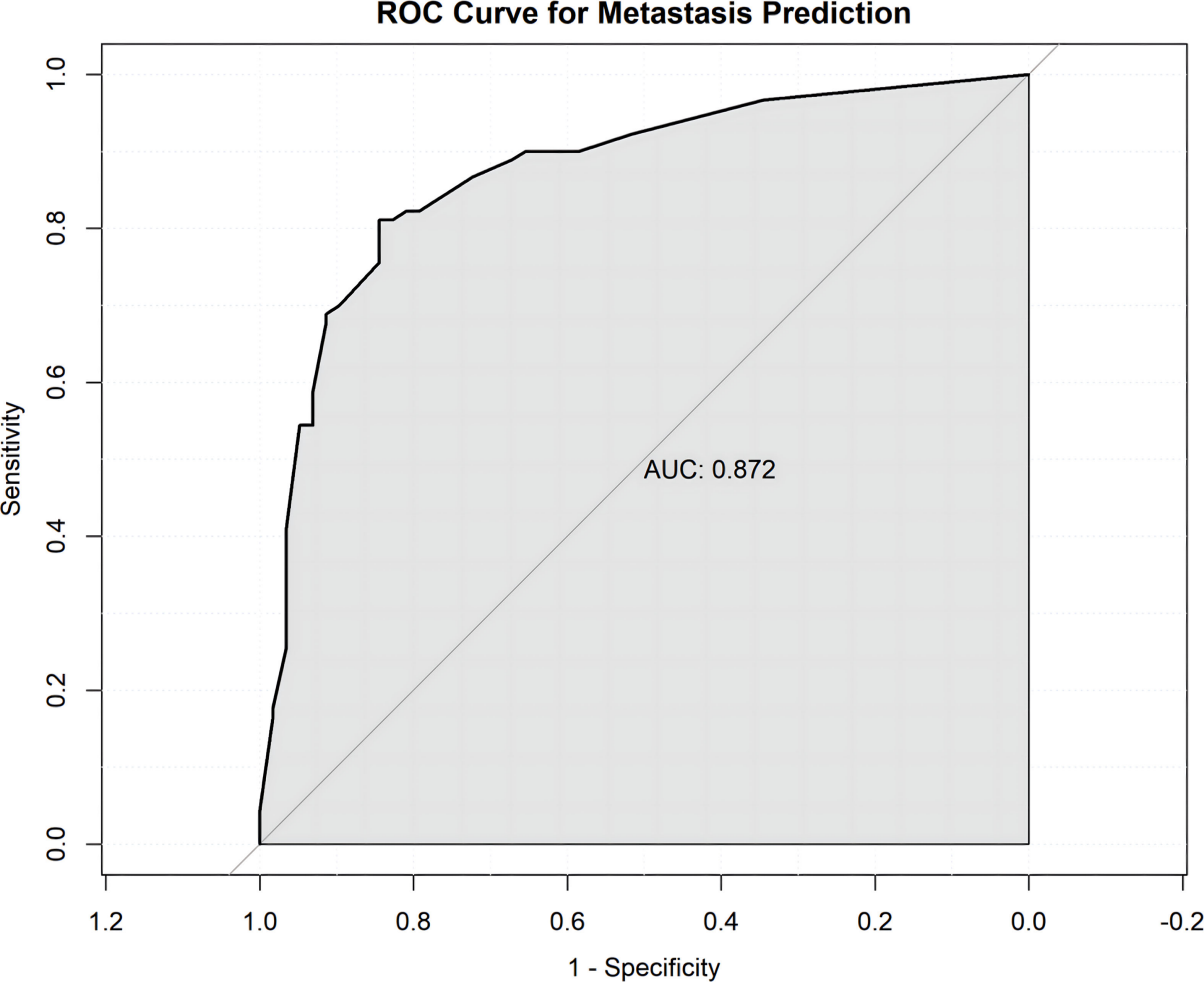
ROC curve for internal validation. ROC curve of the model after bootstrap resampling (1,000 iterations), yielding a calibrated AUC of 0.872.

Internal validation performed through 1000 bootstrap resamples demonstrated good model performance. The calibration plot revealed a high degree of agreement between the predicted and observed probabilities across the entire risk spectrum. The calibrated model’s AUC was 0.872, confirming its ability to discriminate between patients with and without metastasis (**Figures 4**, **5**).

### 3.4 Decision curve analysis

**Figure 6:**
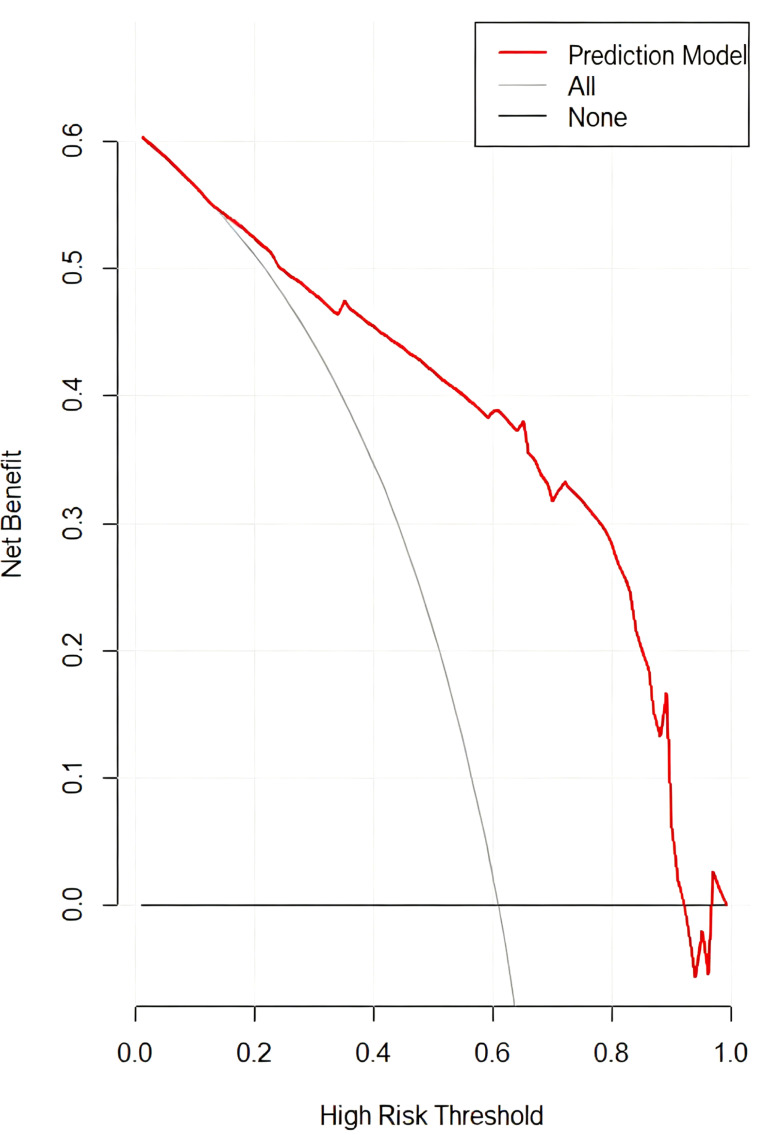
Decision curve analysis (DCA). The DCA indicates that the predictive model provides a positive net benefit across a wide range of threshold probabilities compared to “treat-all” or “treat-none” strategies.

Decision curve analysis reveals that the predictive model demonstrates positive net benefit across the range of threshold probabilities from 0.01 to 0.99. This model demonstrated superior clinical utility for most clinically relevant threshold probabilities compared to the strategies of ‘treat all’ and ‘treat none’(**Figure 6**).

### 3.5 Nomogram construction

**Figure 7:**
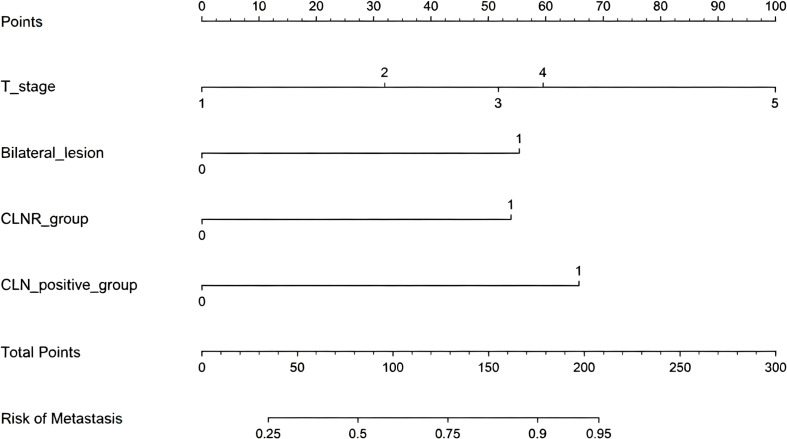
Nomogram for predicting LLNM risk in pediatric DTC. T stage (1 = T1a, 2 = T1b, 3 = T2, 4 = T3, 5 = T4), bilaterality (0=absent, 1=present), CLNR group (0=<0.4, 1=≥0.4), positive CLN count group (0=<4, 1=≥4). Draw a vertical line upward to the Points axis to obtain the score for each predictor. Sum the scores to obtain Total Points, then draw a vertical line downward to the Risk of Metastasis axis.

This nomogram visually presents the metastasis risk prediction model, developed using backward stepwise regression which identified four significant predictors. Each predictor (T stage, bilateral lesion, central LNR group, positive lymph node group) is assigned a score on the top point scale. Draw a vertical line upward to the Points axis to obtain the score for each predictor. Sum the scores to obtain Total Points, then draw a vertical line downward to the Risk of Metastasis axis to estimate the individual probability of LLNM. Using the nomogram, a total point score of ≥250 predicts an LLNM probability >50%, which may be used as a clinical cutoff to guide the decision for prophylactic lateral neck dissection in cN0 pediatric patients with high−risk central node features. The model demonstrates good discriminative ability. This tool provides an intuitive and clinically practical method for metastasis risk stratification(**Figure 7**).

## 4 Discussion

This study, through a retrospective analysis of 148 pediatric DTC patients aged ≤14 years, systematically confirmed for the first time in this specific age group that CLNR ≥0.4 and central positive lymph node count ≥4 are strong and independent predictors of LLNM. These parameters were used to build a predictive model that showed outstanding discrimination (calibrated AUC = 0.872), and decision curve analysis (DCA) confirmed its clinical utility. This finding provides a simple, objective, and quantitative tool for identifying pediatric DTC patients at high risk for LLNM.

### 4.1 The first study to specifically evaluate and validate the central lymph node ratio as a predictor of lateral lymph node metastasis in pediatric patients aged ≤14 years with differentiated thyroid carcinoma

Siegel DA et al. defined childhood cancer as the diagnosis of a first primary malignant tumor in children aged 0–14 years and adolescents aged 15–19 years, based on U.S. Cancer Statistics (USCS) data. This study’s cohort of children under 14 years with differentiated thyroid cancer fills a gap in pediatric cancer research (19).

### 4.2 Comparison with previous studies

In adult DTC, predictive factors for LLNM are relatively well-established, typically including tumor size, extrathyroidal extension, multifocality, and central lymph node metastasis (CLNM) (20). This study also found associations between T stage and bilateral lesions with LLNM in the pediatric population, partially echoing findings from adult studies. However, research specifically focusing on predictors of LLNM in children, particularly younger children (≤14 years), remains scarce. While a study by Kim et al. focused on adolescents ≤18 years and identified predictive value for tumor size, ETE, and multifocal lesions (21), it did not delve into the standardized metric of LNR. This study is the first to introduce CLNR for predicting LLNM in young children and determines its critical cut-off value as 0.4. We hypothesize that due to the often more aggressive lymphatic metastatic tendency of pediatric DTC (2), LNR, which reflects the relative metastatic burden, might better capture its aggressive biological nature compared to some absolute metrics.

### 4.3 Clinical advantages of CLNR ≥0.4 as a predictive indicator

LNR integrates the number of metastatic nodes and the total number of nodes harvested. As a relative ratio, it effectively corrects for biases introduced by variations in the extent of dissection by different surgeons and the number of nodes identified by pathologists, making the assessment more standardized and objective. Rozenblat et al. found that LNR correlated highly with tumor characteristics and prognostic variables in medullary thyroid carcinoma patients (22). Referring to the prognostic value of LNR in other cancers, Hu et al. found that a higher LNR was related to worse disease-free survival in patients diagnosed with thyroid cancer. This suggests that LNR could be a prognostic factor in the future (23).

Study results: In univariate analysis,CLNR ≥0.4 = 90.0% sensitive, 76.0% specific, 77.0% accurate. With an OR of 11.374 (95% CI: 5.177–24.987), it is clearly a strong risk indicator. This clear threshold (0.4) provides an intuitive basis for intraoperative decision-making: when intraoperative frozen section pathology indicates a central LNR reaching or exceeding this value, even if preoperative ultrasound or biopsy did not suggest lateral compartment metastasis, serious consideration should be taken to perform a prophylactic lateral neck dissection concurrently.

### 4.4 The complementary value of LNR and the number of positively tested lymph nodes

The multivariate analysis also showed that the number of ≥4 positive central lymph nodes was an independent predictor. This reflects the importance of absolute metastatic burden. However, the risk information provided by central LNR(relative proportion)and the number of positive nodes(absolute burden)is complementary rather than mutually exclusive. The former reduces procedural variation, while the latter directly reflects disease extent. Taking both into account offers a more thorough description of the lymph node metastatic status. The fact that the AUC of our constructed predictive model (0.877) was significantly higher than any single indicator is the strongest proof. Song et al.’ s study demonstrated that both number ofLNM ≥ 4 and LNR ≥ 0.210 were important prognostic factors for cervical cancer patients (24). Consequently, in clinical practice, it is important to assess both indicators together.

### 4.5 Clinical implications and application prospects

The nomogram developed in this study holds significant potential for clinical translation:

Guiding Surgical Decision-Making: For lateral necks assessed as cN0 preoperatively, if intraoperative frozen section of central lymph nodes indicates a central LNR ≥0.4 or the number of positive nodes ≥4, surgeons can proceed with greater confidence to extend the surgical scope, performing concurrent prophylactic lateral neck dissection (levels II-IV), thereby avoiding secondary surgery.

The optimal treatment strategy remains clinically controversial for patients with preoperative lateral cervical lymph nodes classified as cN0, but whose intraoperative frozen sections revealed a CLNR of ≥0.4 or ≥4 positive central lymph nodes. Decision-making is primarily based on the presence of multiple risk factors:

For patients with two or more high-risk factors (such as higher CLNR, later T-stage, bilateral disease), lateral neck lymph node dissection is typically performed concurrently, as studies have shown that the probability of occult lateral neck metastasis in this subgroup is significantly higher than in patients with a single high-risk factor (25).

For patients with only a single high-risk factor, staged lateral neck dissection may be considered, according to the research by Chu et al., delayed lateral neck lymph node dissection provides similar oncological efficacy as synchronous lateral neck lymph node dissection while reducing the risk of vocal cord paralysis (26), with monitoring via intermittent neck ultrasound. If no evidence of metastasis is found during follow-up, unnecessary lateral neck dissection can be avoided.

For patients with borderline risk factors (such as CLNR slightly above 0.4 but fewer than 4 positive lymph nodes and low T-stage), close monitoring without immediate lateral neck lymph node dissection may be appropriate, reserving radioactive iodine (RAI) therapy for those who show evidence of persistent or recurrent disease. According to the Korean Thyroid Association pediatric guideline, RAI therapy is recommended for iodine-avid pulmonary metastasis and inoperable locoregional lesions (27).

This individualized strategy strikes a balance between the goal of achieving complete tumor resection and the complications associated with lateral neck lymph node dissection, particularly in the pediatric patient population.

It is important to note that the predictive value of CLNR depends on the adequacy of central neck dissection. In patients with a low total number of harvested central lymph nodes (e.g., <5), an CLNR ≥0.4 may reflect a relatively small absolute metastatic burden. In such cases, the decision to perform prophylactic lateral neck dissection should be individualized, considering other risk factors such as T stage, bilaterality, and the absolute number of positive central nodes. The nomogram presented in this study integrates multiple predictors and may provide a more balanced assessment than any single indicator alone.

### 4.6 Optimizing postoperative management

For patients with postoperative paraffin pathology confirming central LNR ≥0.4 or number of positive nodes ≥4, even if the lateral neck was not dissected or was found negative upon dissection, they should be considered at very high risk for recurrence. More intensive postoperative monitoring (e.g., more frequent ultrasound examinations) and discussion regarding the necessity of adjuvant RAI therapy are recommended.

### 4.7 Refining risk stratification

De et al. emphasized the importance of careful risk stratification in pediatric DTC, identifying T4 tumors, WBS lymph node uptake, and gross ETE as independent factors associated with persistent disease in DTC patients under 18 years through long-term follow-up (28). CLNR can serve as an important supplement to the pediatric thyroid cancer risk stratification system, making stratification more precise and treatment strategies more individualized.

### 4.8 Limitations and future directions

Several limitations presented of this study include: (1) This is a single-center study that was conducted retrospectively, which inherently carries selection bias. (2) The sample size is relatively limited. (3) The extent of CND and the number of lymph nodes retrieved may vary among surgeons, although we attempted to correct for this using the LNR metric. (4) The analysis could not examine the relationship between LNR and long-term recurrence rates or disease-free survival; a longer follow-up period is required to validate. (5) Although gene mutation results were considered during initial data collection design, excessive missing values necessitated their exclusion from the analysis. (6) The study conclusions require external validation. (7) The cut-off values determined in this study (0.4 and 4) still need validation in larger, multi-center prospective studies or external datasets.

Although this is a single-center retrospective study, we performed internal validation using the bootstrap method, which enhances the reliability of our conclusions to some extent. Yet the model still requires further validation with external data.

## 5 Conclusion

This study validates that in pediatric differentiated thyroid carcinoma, a CLNR of at least 0.4 and at least 4 metastatic lymph nodes are strong predictors of lateral lymph node metastasis. The simple risk assessment tool constructed based on these two indicators demonstrates excellent performance, aiding in the individualization of surgical decision-making.

We recommend including central LNR, as well as the number of positive lymph nodes, in the preoperative and intraoperative evaluation process for pediatric thyroid cancer, as they provide crucial insights for developing tailored surgical plans and postoperative management strategies. This aims to achieve tumor eradication while minimizing unnecessary surgical trauma and subsequent treatment risks, ultimately improving the long-term quality of physical and mental development in pediatric patients.

## Data Availability

The raw data supporting the conclusions of this article will be made available by the authors, without undue reservation.

## References

[1] Zanella AB Scheffel RS Weinert L Dora JM Maia AL . New insights into the management of differentiated thyroid carcinoma in children and adolescents (Review). Int J Oncol. (2021) 58 (8):13. doi: 10.3892/ijo.2021.5193. PMID: 33649842

[2] Haugen BR Alexander EK Bible KC Doherty GM Mandel SJ Nikiforov YE . 2015 american thyroid association management guidelines for adult patients with thyroid nodules and differentiated thyroid cancer: the american thyroid association guidelines task force on thyroid nodules and differentiated thyroid cancer. Thyroid. (2016) 26:1–133. doi: 10.1089/thy.2015.0020. PMID: 26462967 PMC4739132

[3] Su Y Cheng S Diao C Ma Y Qian J Cheng R . Surgical treatment of pediatric and adolescent papillary thyroid cancer: a retrospective study of 54 patients in a single center. J Pediatr (Rio J). (2022) 98:425–30. doi: 10.1016/j.jped.2021.11.011. PMID: 35139341 PMC9432047

[4] Christison-Lagay E Baertschiger RM . Management of differentiated thyroid carcinoma in pediatric patients. Surg Oncol Clin N Am. (2021) 30:235–51. doi: 10.1016/j.soc.2020.11.013. PMID: 33706898

[5] Zhang X Li JG Zhang SZ Chen G . Comparison of indocyanine green and carbon nanoparticles in endoscopic techniques for central lymph nodes dissection in patients with papillary thyroid cancer. Surg Endosc. (2020) 34:5354–9. doi: 10.1007/s00464-019-07326-4. PMID: 31907662

[6] Ahn JH Kwak JH Yoon SG Yi JW Yu HW Kwon H . A prospective randomized controlled trial to assess the efficacy and safety of prophylactic central compartment lymph node dissection in papillary thyroid carcinoma. Surgery. (2022) 171:182–9. doi: 10.1016/j.surg.2021.03.071. PMID: 34391573

[7] Huang J Li Z Zhong Q Fang J Chen X Zhang Y . Developing and validating a multivariable machine learning model for the preoperative prediction of lateral lymph node metastasis of papillary thyroid cancer. Gland Surg. (2023) 12:101–9. doi: 10.21037/gs-22-741. PMID: 36761483 PMC9906091

[8] Huang H Xu S Ni S Wang X Liu S . A nomogram for predicting lateral lymph node metastasis in cN0 unifocal papillary thyroid microcarcinoma. BMC Cancer. (2023) 23:718. doi: 10.1186/s12885-023-11219-0. PMID: 37528388 PMC10391989

[9] Pino A Mazzeo C Frattini F Zhang D Wu CW Zanghì G . Lymph node dissection morbidity in thyroid cancer: an integrative review. Sisli Etfal Hastan Tip Bul. (2021) 55:433–7. doi: 10.14744/semb.2021.33401. PMID: 35317379 PMC8907691

[10] Pham MC Nguyen T Nguyen HP . Surgical complications in papillary thyroid cancer patients with cervical lymph node metastases. Clin Med Insights Oncol. (2024) 18:11795549241233692. doi: 10.1177/11795549241233692. PMID: 38482163 PMC10935751

[11] van Rooijen JJ van Trotsenburg ASP van de Berg DJ Zwaveling-Soonawala N Nieveen van Dijkum EJM Engelsman AF . Complications after thyroidectomy in children: lymph node dissection is a risk factor for permanent hypocalcemia. Front Endocrinol (Lausanne). (2021) 12:717769. doi: 10.3389/fendo.2021.717769. PMID: 34659111 PMC8511766

[12] Liu W Zhang D Jiang H Peng J Xu F Shu H . Prediction model of cervical lymph node metastasis based on clinicopathological characteristics of papillary thyroid carcinoma: a dual-center retrospective study. Front Endocrinol (Lausanne). (2023) 14:1233929. doi: 10.3389/fendo.2023.1233929. PMID: 37766691 PMC10519787

[13] Tian D Li X Jia Z . Analysis of risk factors and risk prediction for cervical lymph node metastasis in thyroid papillary carcinoma. Cancer Manag Res. (2024) 16:1571–85. doi: 10.2147/cmar.s485708. PMID: 39555445 PMC11566207

[14] Liang W Sheng L Zhou L Ding C Yao Z Gao C . Risk factors and prediction model for lateral lymph node metastasis of papillary thyroid carcinoma in children and adolescents. Cancer Manag Res. (2021) 13:1551–8. doi: 10.2147/cmar.s295420. PMID: 33623434 PMC7896733

[15] Qiu C Wu S Li J . Central lymph node ratio is an important recurrence prognostic factor for pediatric differentiated thyroid cancer. Front Endocrinol (Lausanne). (2024) 15:1290617. doi: 10.3389/fendo.2024.1290617. PMID: 39015179 PMC11250549

[16] Zhou L Gao C Li H Liang W Zeng Q Chen B . Isthmic papillary thyroid carcinoma presents a unique pattern of central lymph node metastasis. Cancer Manag Res. (2020) 12:3643–50. doi: 10.2147/cmar.s252692. PMID: 32547201 PMC7245435

[17] Sheng L Shi J Han B Lv B Li L Chen B . Predicting factors for central or lateral lymph node metastasis in conventional papillary thyroid microcarcinoma. Am J Surg. (2020) 220:334–40. doi: 10.1016/j.amjsurg.2019.11.032. PMID: 31818425

[18] Jeon YW Ahn YE Chung WS Choi HJ Suh YJ . Radioactive iodine treatment for node negative papillary thyroid cancer with capsular invasion only: results of a large retrospective study. Asia Pac J Clin Oncol. (2016) 12:e167–73. doi: 10.1111/ajco.12159. PMID: 24289279

[19] Siegel DA King JB Lupo PJ Durbin EB Tai E Mills K . Counts, incidence rates, and trends of pediatric cancer in the United States, 2003-2019. J Natl Cancer Inst. (2023) 115:1337–54. doi: 10.1093/jnci/djad115. PMID: 37433078 PMC11018256

[20] So YK Kim MJ Kim S Son YI . Lateral lymph node metastasis in papillary thyroid carcinoma: a systematic review and meta-analysis for prevalence, risk factors, and location. Int J Surg. (2018) 50:94–103. doi: 10.1016/j.ijsu.2017.12.029. PMID: 29329789

[21] Kim J Sun Z Adam MA Adibe OO Rice HE Roman SA . Predictors of nodal metastasis in pediatric differentiated thyroid cancer. J Pediatr Surg. (2017) 52:120–3. doi: 10.1016/j.jpedsurg.2016.10.033. PMID: 27836371

[22] Rozenblat T Hirsch D Robenshtok E Grozinsky-Glasberg S Gross DJ Mazeh H . The prognostic value of lymph node ratio in medullary thyroid carcinoma: a multi-center study. Eur J Surg Oncol. (2020) 46:2023–8. doi: 10.1016/j.ejso.2020.04.016. PMID: 32389525

[23] Hu Y Wang Z Dong L Zhang L Xiuyang L . The prognostic value of lymph node ratio for thyroid cancer: a meta-analysis. Front Oncol. (2024) 14:1333094. doi: 10.3389/fonc.2024.1333094. PMID: 38384804 PMC10879587

[24] Song T Chen X Chen F Wan Q Huang W Zhang C . Unraveling the prognostic puzzle: in-depth exploration of lymph node metrics for surgically treated FIGO stage IB-IIA cervical cancer-focus on the number of positive lymph nodes and the lymph node ratio. Int J Surg. (2025) 111 (11):8141–8. doi: 10.1097/js9.0000000000003033. PMID: 40717572 PMC12626453

[25] Fei Y Wang B Yao X Wu J . Factors associated with occult lateral lymph node metastases in patients with clinically lymph node negative papillary thyroid carcinoma: a systematic review and meta-analysis. Front Endocrinol (Lausanne). (2024) 15:1353923. doi: 10.3389/fendo.2024.1353923. PMID: 39493782 PMC11527613

[26] Chu F De Berardinis R Tagliabue M Bruschini R Zorzi SF Manzoni MF . Surgical timing in thyroid cancer with lateral neck metastases: delayed versus contemporary lateral neck dissection. Cancers (Basel). (2025) 17 (16):2649. doi: 10.3390/cancers17162649. PMID: 40867278 PMC12384942

[27] Lee EK Lee YA . Pediatric thyroid cancer: key considerations based on the 2024 Korean Thyroid Association Differentiated Thyroid Cancer Management Guidelines. Ann Pediatr Endocrinol Metab. (2025) 30:48–51. doi: 10.6065/apem.2448296.148. PMID: 39757597 PMC11917397

[28] De Leo S Bottici V Pellegriti G Russo M Mian C Vianello F . Clinical and pathological factors associated with disease persistence in pediatric patients with differentiated thyroid carcinoma. Thyroid. (2025) 35:1013–23. doi: 10.1177/10507256251363978. PMID: 40737224

